# 20(S/R)-Ginsenoside Rh1 Alleviates AOM/DSS-Induced Colorectal Cancer: Gut-Microbiota Modulation and Tryptophan-Metabolism-Mediated AhR/PXR Activation and IDO1

**DOI:** 10.3390/ijms27125477

**Published:** 2026-06-17

**Authors:** Linqian Lu, Jinyu Min, Yansong Gao, Ge Yang, Zijian Zhao, You Kang, Yujuan Zhao, Lei Zhao, Shengyu Li

**Affiliations:** 1 School of Pharmaceutical Sciences, Changchun University of Chinese Medicine, Changchun 130117, China; 15066094654@163.com (L.L.); mjy08018188@163.com (J.M.); 2 Institute of Agro-Food Technology, Jilin Academy of Agricultural Sciences (Northeast Agricultural Research Center of China), Changchun 130033, China; gysgerry@126.com (Y.G.); yangge1900@163.com (G.Y.); zhaojaas@163.com (Z.Z.); kangkang.1982@163.com (Y.K.); sunny3173@126.com (Y.Z.)

**Keywords:** 20(S/R)-ginsenoside Rh1, colorectal cancer, gut microbiota, tryptophan metabolism, intestinal barrier protection, anti-tumor immunity

## Abstract

Colorectal cancer (CRC) is intricately linked to gut microbiota dysbiosis and tryptophan (Trp) metabolic dysregulation. This study aimed to clarify the role and mechanisms of 20(S/R)-ginsenoside Rh1 in suppressing colorectal cancer through the regulation of gut microbiota and Trp metabolism. Azoxymethane/dextran sulfate sodium (AOM/DSS)was employed to induce a CRC mouse model, followed by treatment with 20(S/R)-ginsenoside Rh1 at 100 mg·kg^−1^·day^−1^ for 6 weeks. 20(S/R)-ginsenoside Rh1 significantly reduced the disease activity index (DAI) score, restored colon length, and decreased tumor count. 20(S/R)-Ginsenoside Rh1 ameliorated gut dysbiosis by increasing gut microbial diversity and elevating the prevalence of beneficial bacteria, including *Lactobacillus*, and stimulated the production of indole derivatives, including indole-3-propionic acid (IPA), indole-3-acetic acid (IAA), and indole-3-lactic acid (ILA) by enriching Trp -metabolizing bacteria such as *Lactobacillus reuteri*. These changes further activated the AhR/CYP1A1/IL-22 and PXR/TLR4 pathways, upregulated the expression of intestinal tight junction proteins, suppressed the secretion of proinflammatory cytokines, including tumor necrosis factor-alpha (TNF-α), interleukin-6 (IL-6), and IFN-γ, and elevated the levels of the anti-inflammatory cytokine IL-10. Furthermore, 20(S/R)-ginsenoside Rh1 reduces the serum kynurenine (Kyn)/Trp ratio, downregulates the expression of forkhead box P3 (FoxP3), a marker of regulatory T (Treg) cells, and increases the number of CD8^+^ T cells by inhibiting the expression of indoleamine 2,3-dioxygenase 1 (IDO1) in colonic tissue. In conclusion, 20(S/R)-ginsenoside Rh1 showed potential anti-CRC activity, with our study observing links between its action and gut microbiota structure regulation, Trp metabolism modulation, AhR/PXR-mediated intestinal barrier activation, and IDO1-related immune suppression reversal.

## 1. Introduction

Worldwide, colorectal cancer (CRC) is a malignant tumor with a high incidence and fatality rate [1]. Unlike other malignancies, CRC interacts directly with trillions of gut microorganisms during tumorigenesis and progression. The composition of the intestinal flora is regulated by various factors such as diet, drugs, and genetic variations, and changes in the flora structure can induce dysbiosis and promote the development of CRC [2]. Research has revealed that intestinal flora disorder occurs in the very early stage of CRC tumor formation and further aggravates with disease progression [3]. Furthermore, microbiota-derived metabolites (e.g., tryptophan (Trp), short-chain fatty acids) may exert either promotional or inhibitory effects on CRC initiation and progression [4,5].

Trp metabolism in the intestine is carried out through the intestinal flora-mediated indole pathway and the host’s kynurenine (Kyn) pathway, which is crucial for host health [6]. Studies on intestinal flora metabolites have found that indole levels in fecal samples of CRC patients are lower compared with those of healthy people [7]. By binding to the aryl hydrocarbon receptor (AhR) and pregnane X receptor (PXR), indole derivatives can improve tight junctions and the function of the intestinal barrier, and control gut immunological tolerance [8]. Among them, AhR exerts intestinal barrier protection function by enhancing the expression of interleukin-22 (IL-22) to prevent intestinal inflammation and the development of CRC [9]. It has also been found that indole-3-lactic acid (ILA) can regulate macrophage differentiation by activating AhR in macrophages, maintain intestinal homeostasis, and thus play an anti-CRC role [10]. Indole-3-propionic acid (IPA), as a ligand of PXR, protects intestinal barrier integrity by down-regulating tumor necrosis factor-alpha (TNF-α) in intestinal cells, up-regulating junction protein-encoding mRNA, and regulating it through the Toll-like receptor 4 (TLR4) signaling pathway [11]. Furthermore, PXR may exert chemopreventive effects on inflammation-induced CRC via mediating inflammation-alleviating, cellular proliferation-inhibiting, and apoptosis-inducing effects [12]. Kyn concentrations in fecal samples from CRC patients are significantly elevated compared to those observed in healthy individuals [7]. This phenomenon is mainly attributed to the high expression of indoleamine 2,3-dioxygenase 1 (IDO1) in various cancer types, including CRC [13]. IDO1 catalyzes the decomposition of Trp in the Kyn pathway, leading to Trp depletion and Kyn production [14]. Kyn is considered a pro-cancer metabolite in CRC. Kyn promotes the development of CRC through multiple mechanisms, including promoting regulatory T cell (Tregs) differentiation, CD8^+^ T cell exhaustion, increasing CRC cell proliferation, and inhibiting apoptosis [15]. The Trp metabolic pathway, by mediating the interactions among gut microbiota, host immune response, and tumor cell signaling pathways, serves as a key regulatory node in the progression of CRC from precancerous lesions to malignancy. Its metabolic imbalance can directly induce the formation of an intestinal immunosuppressive microenvironment and promote tumor cell proliferation and invasion [16,17]. Therefore, the development of natural products that can restore intestinal flora to increase indole derivatives and inhibit IDO1 and Kyn production may provide a new and effective way for the prevention and treatment of CRC.

Ginsenosides, the primary bioactive components of ginseng, are reported to exhibit diverse pharmacological activities, including cancer-suppressive, senescence-inhibiting, inflammation-alleviating, diabetes-mitigating, neuron-protective, and cardioprotective effects [18]. In particular, ginsenoside Rh1 has an effective inhibitory effect on the growth of various tumor types. In liver cancer models, ginsenoside Rh1 upregulates major histocompatibility complex class-I expression by inhibiting glucocorticoid receptors, improves the immunosuppressive tumor microenvironment, and thus exerts anti-tumor effects [19]; in breast cancer, it may exert suppressive effects on cancer cell growth, migration, and invasion via diverse pathways [20,21,22]; in gastric cancer studies, there is also evidence that it exerts anti-cancer effects by targeting the TGF-β/Smad pathway [23]; furthermore, in CRC, ginsenoside Rh1 may sup-press cancer cell proliferative capacity, migratory potential, and invasive capability by downregulating the expression of matrix metalloproteinases 1 and 3, upregulating the expression of tissue inhibitor of metalloproteinase 3, and attenuating the mitogen-activated protein kinase signaling pathway [24]. Given the critical role of the Trp metabolic pathway in maintaining intestinal homeostasis and regulating the immune microenvironment of CRC, combined with the reported activity of ginsenoside Rh1 in remodeling the immune microenvironment, as well as the research basis of other ginsenosides in improving gut microbiota and metabolic disorders and preventing CRC [25], it is speculated that this pathway may be a potential target of ginsenoside Rh1 against CRC. However, there is no research report on ginsenoside Rh1 inhibiting CRC by regulating intestinal flora and Trp metabolism. This study established a CRC model using azoxymethane/dextran sulfate sodium (AOM/DSS) to investigate the effects of 20(S/R)-ginsenoside Rh1 on the regulation of intestinal microbiota, Trp and its metabolites, thereby activating AhR/PXR and inhibiting IDO1 expression, and ultimately inhibiting CRC.

## 2. Results

### 2.1. 20(S/R)-Ginsenoside Rh1 Treatment Inhibits Tumor Progression in CRC Mice

**Figure 1:**
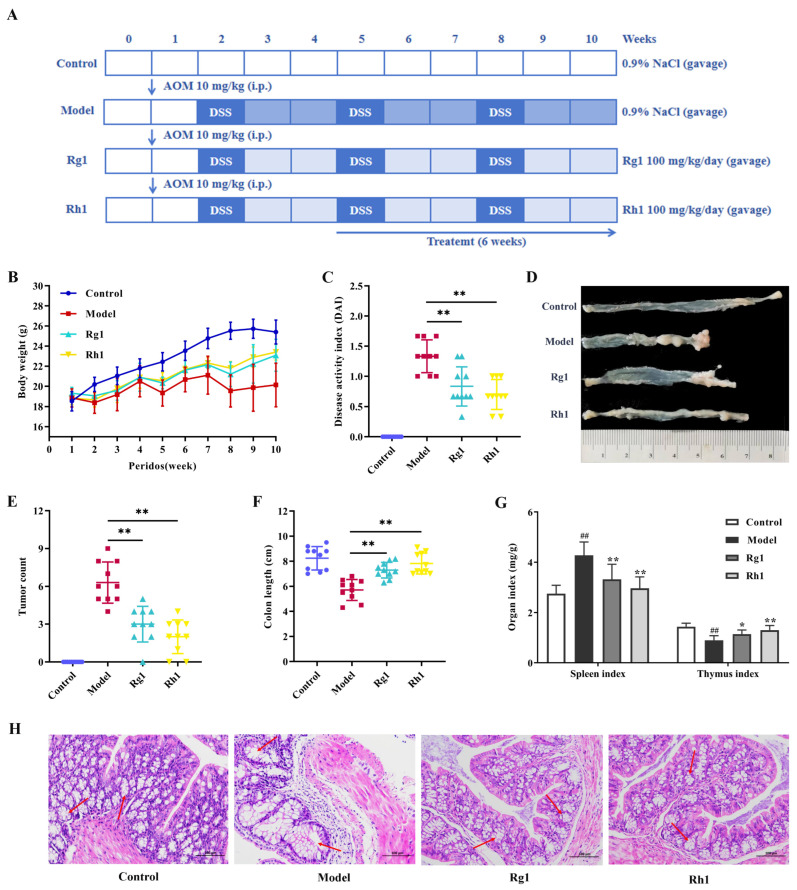
20(S/R)-Ginsenoside Rh1 ameliorates AOM/DSS-induced CRC. (**A**) Flow chart of modeling and drug administration. (**B**) Body weight. (**C**) The disease activity index (DAI) score was measured. (**D**) Longitudinal section of colon. (**E**) Tumor count. (**F**) Colon lengths. (**G**) Organ index. (**H**) Representative hematoxylin and eosin (H&E)-stained images of mouse colon tissue (200× magnification). Arrows indicate crypt architectural distortion, epithelial dysplasia, and inflammatory infiltration. Data are presented as mean ± SD. ^##^ *p* < 0.01 versus the control group; * *p* < 0.05, ** *p* < 0.01 versus the model group (*n* = 10).

As shown in Figure 1B, mice receiving 20(S/R)-ginsenoside Rh1 maintained a body weight significantly higher than that of the model group (*p* < 0.01). Compared to the model group, the disease activity index (DAI) score fell from 1.33 ± 0.27 to 0.83 ± 0.32 in the Rg1 group and further to 0.70 ± 0.24 in the Rh1 group, with the Rh1 group value significantly lower than that of the Rg1 group (*p* < 0.01, Figure 1C). Tumor count in the Rh1 group was significantly reduced to 2.00 ± 1.33, compared with 6.30 ± 1.64 in the model group (*p* < 0.01, Figure 1D,E). Colon shortening observed in the model group was significantly reversed after 20(S/R)-ginsenoside Rh1 administration, restoring length to values comparable to the control (*p* < 0.01, Figure 1F). Figure 1G shows that 20(S/R)-ginsenoside Rh1 treatment almost completely normalized the spleen index and restored the thymus index to near-control levels, outperforming Rg1 in both parameters (*p* < 0.01). hematoxylin and eosin (H&E) histological analysis (Figure 1H) revealed that 20(S/R)-ginsenoside Rh1 treatment preserved crypt architecture, reduced epithelial dysplasia, and diminished inflammatory infiltration, thereby attenuating the hallmark features of AOM/DSS-induced CRC.

### 2.2. 20(S/R)-Ginsenoside Rh1 Ameliorates Gut Microbiota Dysbiosis in CRC Mice

**Figure 2:**
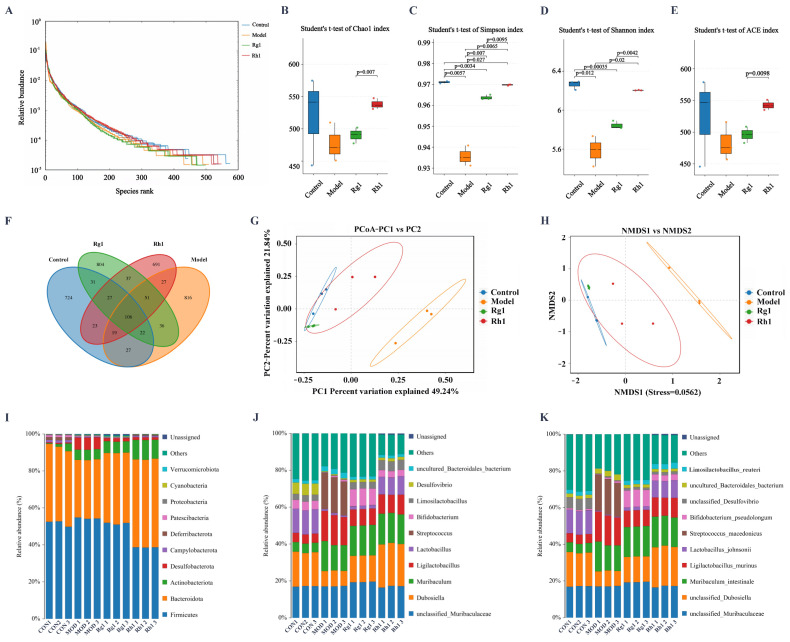
Effects of 20(S/R)-Ginsenoside Rh1 on gut microbiota diversity and community composition in CRC mice. (**A**) Rank-abundance curve. (**B**–**E**) Alpha diversity analysis of the gut microbiota utilizing Chao1 estimator (Chao1), Simpson index, Shannon index, and abundance-based coverage estimator (ACE). (**F**) Overlap analysis of operational taxonomic units (OTU). (**G**,**H**) Beta diversity analysis of the gut microbiota utilizing Principal Coordinates Analysis (PCoA) and Non-metric Multidimensional Scaling (NMDS). (**I**) Relative abundance of intestinal microbiota at the phylum level. (**J**) Relative abundance of intestinal microbiota at the genus level. (**K**) Relative abundance of intestinal microbiota at the species level. Data are presented as mean ± SD (*n* = 3).

**Figure 3:**
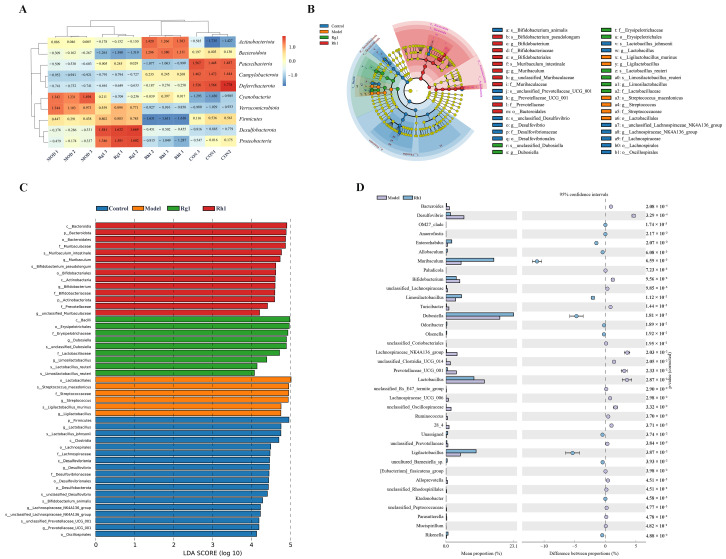
20(S/R)-Ginsenoside Rh1 can induce intergroup differences in the abundance of gut microbiota in CRC mice. (**A**) Phylum-level Species Abundance Cluster Heatmap. (**B**) Analysis of differences in microbiota between groups via Linear Discriminant Analysis (LDA) Effect Size (LEfSe). (**C**) The relative abundance of bacterial taxa was determined via the LEfSe algorithm. Each bar graph denotes the log10 effect size (LDA score > 4) for a given taxon. (**D**) Differential abundance analysis of species at the genus level between samples. Data are presented as mean ± SD (*n* = 3).

The effects of 20(S/R)-ginsenoside Rh1 on intestinal microbiota were analyzed through 16S ribosomal RNA sequencing. Species-rank abundance curves were flat and spanned a wide horizontal range, indicating high richness and evenness across all samples (Figure 2A). Compared to the model and Rg1 groups, the Rh1 group exhibited markedly elevated Chao1 estimator (Chao1), Simpson diversity index, Shannon diversity index, and abundance-based coverage estimator (ACE) indices (*p* < 0.01, Figure 2B–E), reflecting increased species diversity. Venn analysis identified 3441 operational taxonomic units (OTUs), with 106 being common OTUs (Figure 2F). Principal coordinate analysis (PCoA) and Non-metric Multidimensional Scaling (NMDS) revealed a clear shift: the microbial community in the Rh1 group converged toward the profile of the control group (Figure 2G,H). At the phylum level, the model group displayed a reduced relative abundance of *Bacteroidetes* alongside elevated levels of the potentially detrimental phyla *Firmicutes* and *Desulfobacterota* relative to the control group. Conversely, 20(S/R)-ginsenoside Rh1 treatment significantly restored *Bacteroidetes* levels and concurrently suppressed *Firmicutes* and *Desulfobacterota* (Figure 2I). The Rh1 group achieved a greater recovery amplitude of *Bacteroidetes* and better inhibition effect on harmful phyla than the Rg1 group. A phylum-level clustered heatmap further corroborated these findings, showing that 20(S/R)-ginsenoside Rh1 augmented *Bacteroidetes* and *Actinobacteria* while curtailing *Patescibacteria* compared with the model group (Figure 3A). Zooming in to the genus level, Rh1 administration robustly enriched *Dubosiella*, *Muribaculum*, *Lactobacillus*, *Bifidobacterium*, and *Limosilactobacillus*, whereas the opportunistic pathogen *Streptococcus* was conspicuously diminished (Figure 2J). The Rg1 group had a more significant effect on increasing *Bifidobacterium*, but Rh1 performed better in regulating other key genera and improving overall microbial structure stability. At the species level, 20(S/R)-ginsenoside Rh1 significantly increased the abundances of *unclassified-Dubosiella*, *Lactobacillus johnsonii*, and *Lactobacillus reuteri*, while significantly decreasing the abundance of *Streptococcus macedonicus* (Figure 2K). The Rg1 group primarily enhanced *Bifidobacterium pseudolongum*, whereas the Rh1 group showed a better overall optimization effect on microbial functions. Inter-group comparisons underscored the restorative capacity of 20(S/R)-ginsenoside Rh1, as its microbiota composition mimicked that of the control group more closely than any other treatment. Specifically, *Muribaculum*, *Limosilactobacillus*, *Dubosiella*, and *Ligilactobacillus* were significantly more abundant in the Rh1 group than in the model group (Figure 3B–D). These key genera in the Rh1 group were also closer to the control group level with greater recovery amplitude than the Rg1 group.

### 2.3. 20(S/R)-Ginsenoside Rh1 Regulates Serum Trp Metabolism in CRC Mice

**Figure 4:**
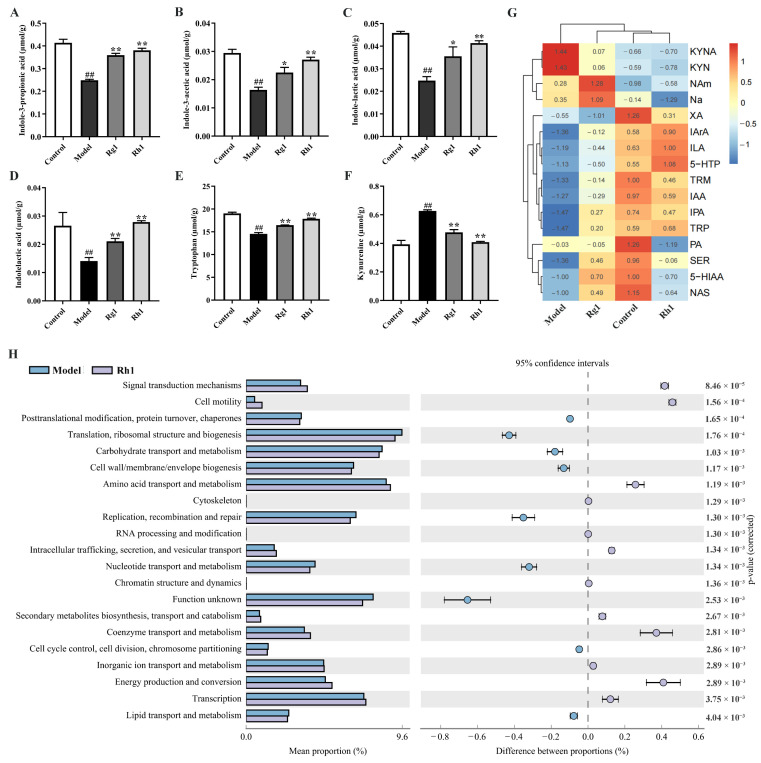
The influence of 20(S/R)-ginsenoside Rh1 on serum Trp metabolism. Content of gut microbiota-associated Trp metabolites (**A**) indole-3-propionic acid, (**B**) indoleacetic acid, (**C**) indole-3-lactic acid, (**D**) indole-3-acrylic acid, (**E**) tryptophan, and (**F**) kynurenine. (**G**) Inter-group correlation analysis of Trp metabolome. (**H**) MetaCyc enrichment analysis. Data are presented as mean ± SD. ^##^ *p* < 0.01 versus the control group; * *p* < 0.05, ** *p* < 0.01 versus the model group (*n* = 3).

Targeted metabolomics was employed to detect the levels of Trp metabolites in mouse serum. Compared with the model group, after treatment with 20(S/R)-ginsenoside Rh1, the levels of indole-3-propionic acid (IPA), indole-3-acetic acid (IAA), indole-3-lactic acid (ILA), and indole-3-acrylic acid (IArA) were all significantly increased (by 53.05%, 65.64%, 66.62%, and 98.06%, respectively; *p* < 0.01, Figure 4A–E). Further comparison of the regulatory effects between the Rh1 group and the Rg1 group revealed that the Rh1 group exerted a more significant promoting effect on the aforementioned indole derivatives: IPA was significantly increased by 23.67% (*p* < 0.01), IArA by 32.23% (*p* < 0.01), IAA by 20.26% (*p* < 0.05), and ILA by 23.60%. In addition to the positive regulation of indole derivatives, as shown in Figure 4F, the levels of kynurenine (Kyn) in both the Rg1 group and Rh1 group were significantly lower than those in the model group (*p* < 0.01); furthermore, the Kyn level in the Rh1 group was further reduced by 14.28% compared with the Rg1 group (*p* < 0.05). The heatmap data showed significant differences in Trp metabolite levels between the model group and the Rh1 group (Figure 4G). In the model group, Kyn and kynurenic acid (KynA) levels were significantly upregulated; the Rh1 group downregulated Kyn and KynA levels while increasing IAA and ILA contents, and compared with the Rg1 group, the metabolite levels in the Rh1 group were closer to those in the control group. MetaCyc enrichment analysis revealed that the differential gut microbiota distinguishing the model group from the Rh1 group are linked to metabolic pathways including amino acid transport and metabolism, carbohydrate transport and metabolism, translation, and ribosomal structure and biogenesis (Figure 4H).

### 2.4. Protective Effect of 20(S/R)-Ginsenoside Rh1 on the Intestinal Barrier of CRC Mice via the AhR/PXR Pathways

**Figure 5:**
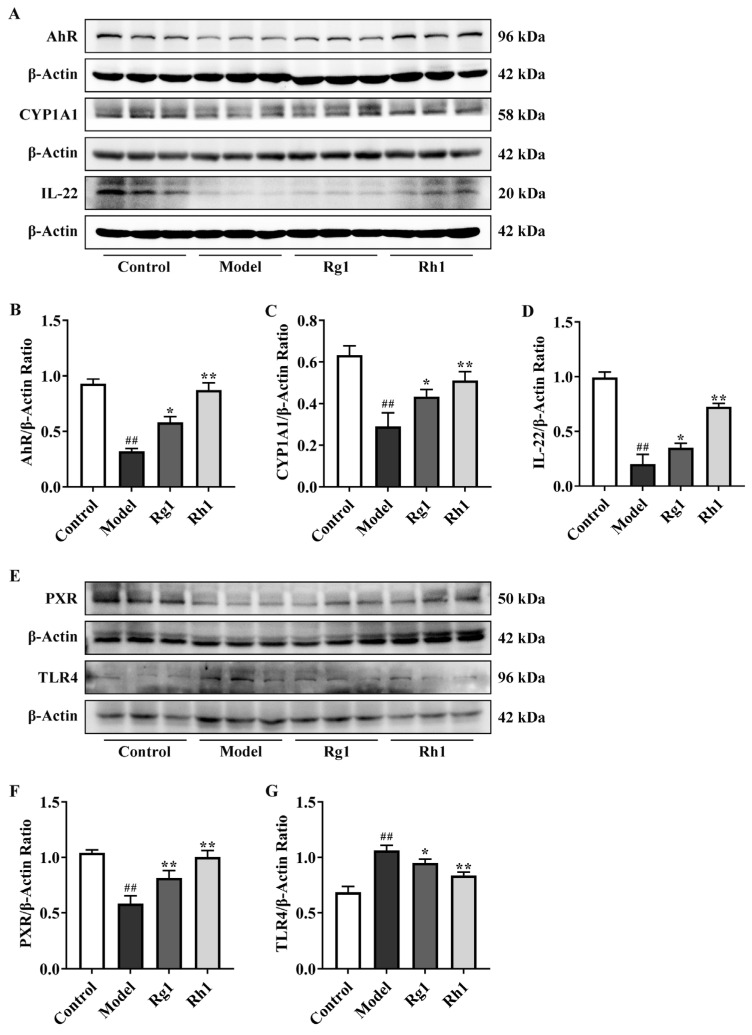
Effects of 20(S/R)-ginsenoside Rh1 on AhR/PXR pathways in CRC mice. (**A**–**D**) Representative Western blot picture and relative protein expression levels of AhR, CYP1A1, IL-22, and β-Actin. (**E**–**G**) Representative Western blot picture and relative protein expression levels of PXR, TLR4, and β-Actin. Data are presented as mean ± SD. ^##^ *p* < 0.01 versus the control group; * *p* < 0.05, ** *p* < 0.01 versus the model group (*n* = 3).

**Figure 6:**
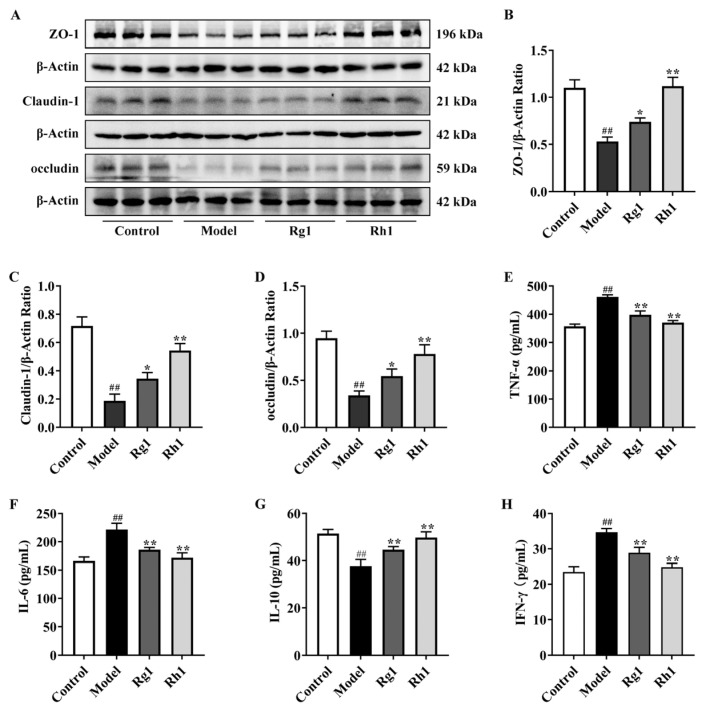
Effects of 20(S/R)-ginsenoside Rh1 on intestinal tight junction protein expression and inflammatory factor levels in CRC mice. (**A**–**D**) Representative Western blot picture and relative protein expression levels of ZO-1, Claudin-1, occludin, and β-Actin. (**E**–**H**) Relative expression levels of serum inflammatory cytokines TNF-α, IL-6, IL-10, and IFN-γ. Data are presented as mean ± SD. ^##^ *p* < 0.01 versus the control group; * *p* < 0.05, ** *p* < 0.01 versus the model group (*n* = 3).

Indole derivatives generated by the gut microbiota are agonists of AhR and PXR, both of which are critical for intestinal barrier integrity. Compared with the model group, the expression levels of AhR, CYP1A1, and IL-22 in the Rh1 group were significantly increased (0.32 ± 0.03 vs. 0.87 ± 0.07; 0.23 ± 0.07 vs. 0.51 ± 0.04; 0.20 ± 0.09 vs. 0.72 ± 0.03; *p* < 0.01, Figure 5A–D). Following treatment with 20(S/R)-ginsenoside Rh1, PXR expression increased by 41.95%, while TLR4 expression decreased significantly by 27.10% (*p* < 0.01, Figure 5E–G). The expression levels of PXR and TLR4 in the Rg1 group tended towards those of the control group, and this effect became more pronounced after treatment with 20(S/R)-ginsenoside Rh1 (*p* < 0.05). Western blot analysis of colonic tissue showed that treatment with 20(S/R)-ginsenoside Rh1 significantly increased ZO-1, Claudin-1, and occludin protein expression by 52.64%, 65.58%, and 56.39%, respectively (*p* < 0.01, Figure 6A–D). Ginsenoside Rg1 also upregulated ZO-1, Claudin-1, and occludin expression (*p* < 0.05), though its effect was less pronounced than 20(S/R)-ginsenoside Rh1. Compared to the model group, 20(S/R)-ginsenoside Rh1 administration significantly decreased TNF-α, interleukin-6 (IL-6), and interferon-gamma (IFN-γ) levels by 19.70%, 20.47%, and 28.44%, respectively, and increased interleukin-10 (IL-10) expression by 31.81% (*p* < 0.01, Figure 6E–H). Collectively, 20(S/R)-ginsenoside Rh1 orchestrates AhR and PXR-mediated signaling to suppress inflammation and reinforce tight junction integrity, thereby safeguarding the colonic barrier in CRC.

### 2.5. 20(S/R)-Ginsenoside Rh1 Altered the Downregulated IDO1 Expression and Kyn/Trp Ratio in CRC Mice

**Figure 7:**
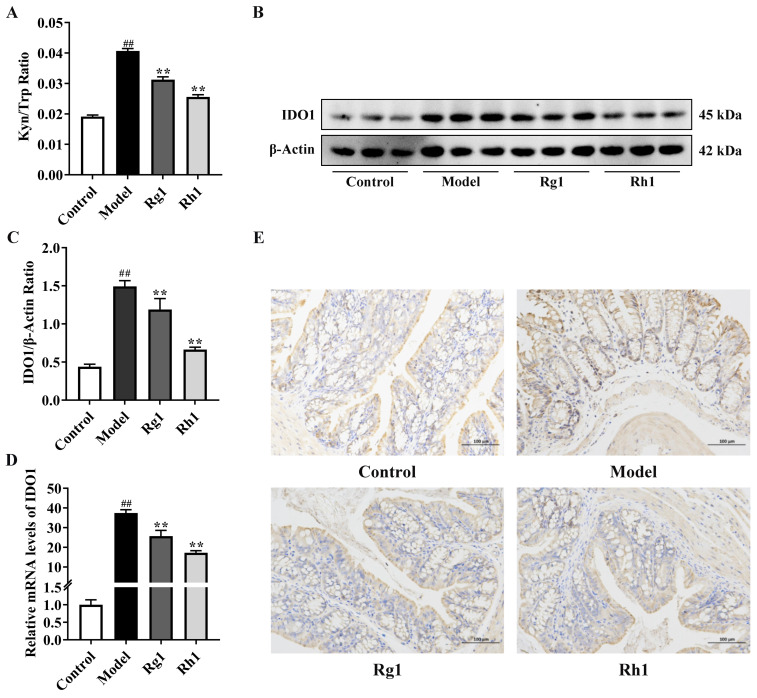
20(S/R)-Ginsenoside Rh1 Modulates Kyn/Trp Ratio and IDO1 Expression in Colon Cancer Mice. (**A**) IDO1 enzyme activity (expressed as Kyn/Trp ratio). (**B**,**C**) Representative Western blot picture and relative protein expression levels of IDO1 and β-Actin. (**D**) Expression of IDO1 mRNA in mouse colon tissues. (**E**) Immunohistochemical analysis of IDO1 expression in mouse colon tissues. Data are presented as mean ± SD. ^##^ *p* < 0.01 versus the control group; ** *p* < 0.01 versus the model group (*n* = 3).

**Figure 8:**
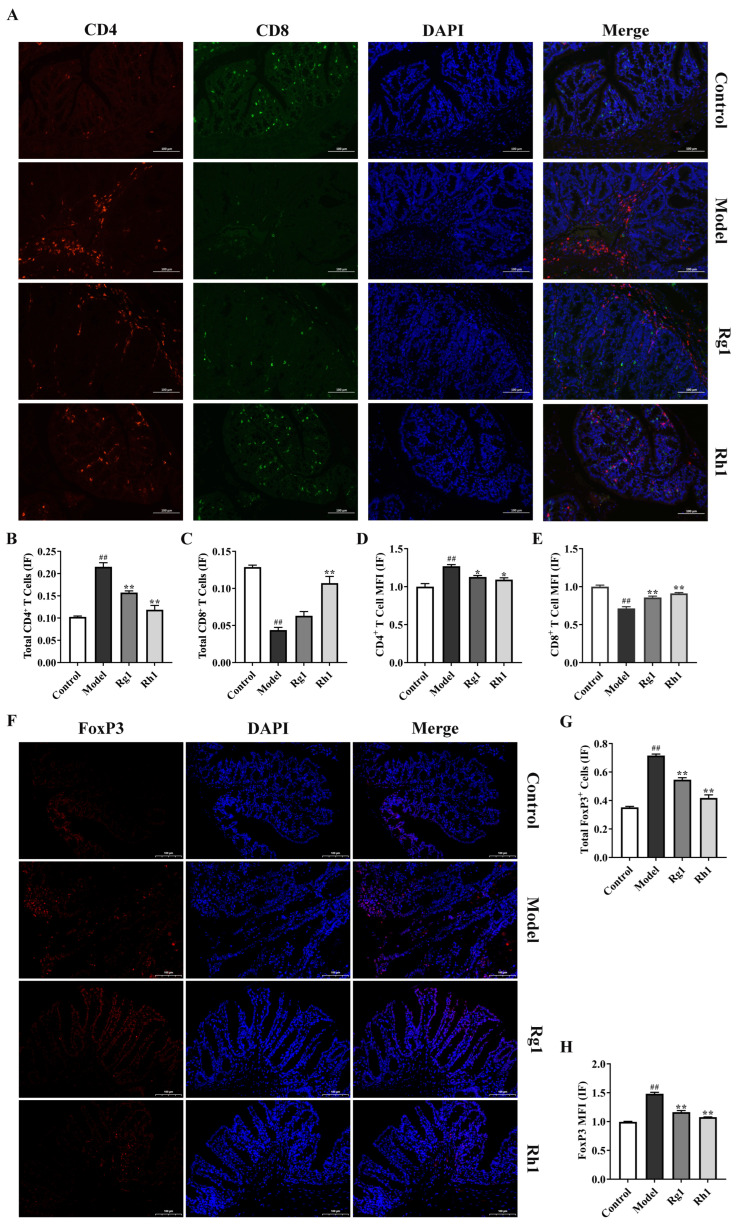
Effects of 20(S/R)-ginsenoside Rh1 on T cells in colon cancer mice. (**A**) Representative immunofluorescence images of CD4^+^ (red) and CD8^+^ (green) T cells in mouse colon tissues. (**B**,**C**) Total number of CD4^+^ and CD8^+^ T cells. (**D**,**E**) Mean fluorescence intensity (MFI) of CD4^+^ and CD8^+^ T cells. (**F**) Representative immunofluorescence images of FoxP3 (red, Treg marker) in mouse colon tissues. (**G**) Total number of FoxP3-positive cells. (**H**) MFI of FoxP3. Nuclei were counterstained with DAPI (blue) in all immunofluorescence panels. Data are presented as mean ± SD. ^##^ *p* < 0.01 versus the control group; * *p* < 0.05, ** *p* < 0.01 versus the model group (*n* = 3).

As shown in Figure 6A, the serum levels of Trp decreased while those of Kyn increased in the model group, resulting in an elevated Kyn/Trp ratio. Western blot results show that 20(S/R)-ginsenoside Rh1 reduced the expression of IDO1 in mouse colon tissue (Figure 7B,C). To further verify the expression of the *IDO1* gene, we examined the mRNA levels of *IDO1* in colon tissue. The elevated *IDO1* mRNA expression was observed in the model group, whereas the Rh1 group showed a significant 32.91% downregulation (*p* < 0.01, Figure 7D). Immunohistochemical staining also yielded similar results (Figure 7E). Immunofluorescence staining results showed that in the colonic tumor tissues of mice in the model group, the level of CD8^+^ T cells was significantly decreased, while the number of CD4^+^ T cells, as well as the total number of positive cells and immunofluorescence intensity of forkhead box P3 (FoxP3, a marker of Treg cells), were significantly increased. After treatment with 20(S/R)-ginsenoside Rh1, the level of CD8^+^ T cells was significantly restored, the number of CD4^+^ T cells was significantly reduced, and meanwhile, both the total number of positive cells and immunofluorescence intensity of FoxP3 were significantly decreased compared with the model group (*p* < 0.01, Figure 8A–H).

### 2.6. Correlation Analysis of Gut Microbiota, CRC-Related Parameters, and Trp and Its Metabolites

**Figure 9:**
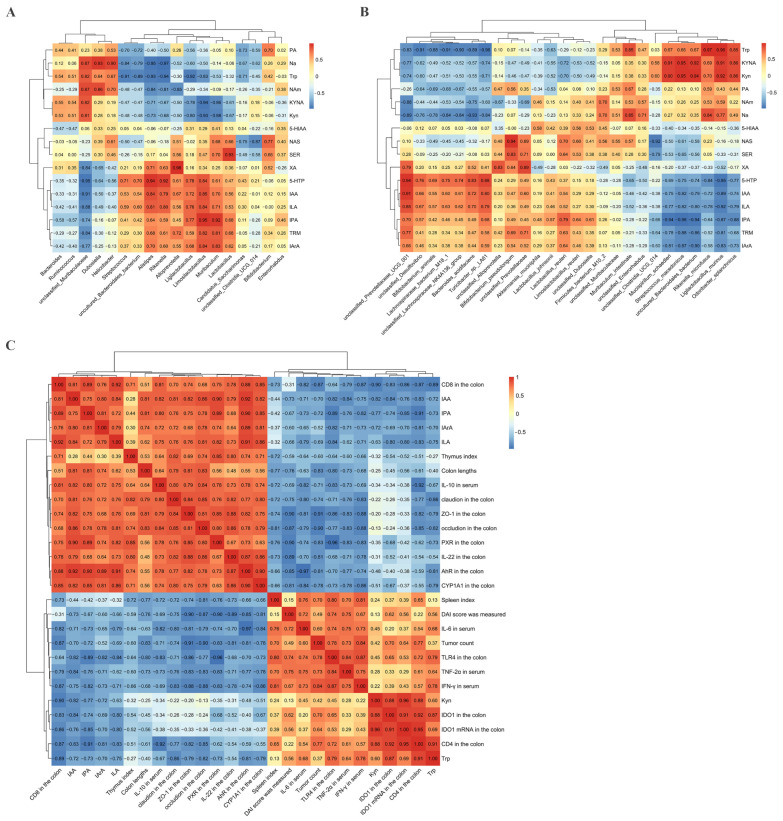
Correlation analysis of gut microbiota, CRC-related parameters, and Trp and its metabolites. (**A**) Correlation analysis results between gut microbiota at the genus level and Trp and its metabolites. (**B**) Correlation analysis results between gut microbiota at the species level and Trp and its metabolites. (**C**) Correlation analysis results between CRC-related parameters and Trp and its metabolites. Data are presented as mean ± SD (*n* = 3).

Pearson correlation analysis was performed to assess associations between altered gut microbiota, CRC-related parameters, and Trp metabolites. As shown in Figure 9A, the correlation analysis between gut microbiota at the genus level and Trp metabolism showed that *Lactobacillus*, *Limosilactobacillus*, *Muribaculaceae*, and *Ligilactobacillus* correlated positively with indole derivatives such as IPA, ILA, and IAA and negatively correlated with Kyn. Analysis at the species level (Figure 9B) showed that *Ligilactobacillus murinus*, *Lactobacillus johnsonii*, and *Limosilactobacillus reuteri* correlated positively with IPA, IAA, ILA, and IArA, while *Streptococcus macedonicus* correlated positively with Kyn and KynA and negatively correlated with IPA. The correlation analysis between CRC-related parameters and Trp metabolites in Figure 9C revealed significant synergistic correlations among indole derivatives. Specifically, their concentrations were positively associated with colon length, serum anti-inflammatory factor IL-10, colonic barrier markers (ZO-1, occludin, claudin). Meanwhile, they were also significantly positively correlated with key molecules of the AhR/PXR signaling pathway (AhR, CYP1A1, IL-22, PXR), while negatively associated with tumor burden, serum pro-inflammatory factors (TNF-2α, IL-6, IFN-γ), and the expression of TLR4 and IDO1 in the colon. In addition, Kyn was significantly positively correlated with IDO1 enzyme activity and CD4^+^ T cell infiltration, but significantly negatively correlated with indole derivatives and CD8^+^ T cell levels.

## 3. Discussion

**Figure 10:**
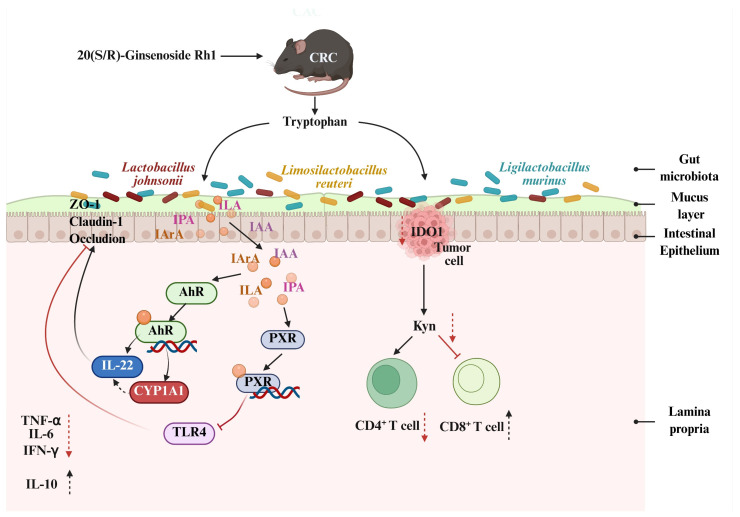
Mechanism of action. 20(S/R)-Ginsenoside Rh1 exerts anti-colorectal cancer effects by regulating gut microbiota, promoting the production of indole derivatives such as IPA and IAA to activate the AhR/CYP1A1/IL-22 and PXR/TLR4 pathways, enhancing intestinal barrier function and inhibiting inflammation; meanwhile, it inhibits colonic IDO1 expression, reduces the serum Kyn/Trp ratio, increases the proportion of CD8^+^ T cells, decreases the number of CD4^+^ T cells. Red arrows indicate inhibition or decrease; black arrows indicate promotion or increase.

Studies have demonstrated that the gut microbiota is closely associated with the pathogenesis of CRC, and Ginsenosides can modulate the development of CRC by regulating the gut microbiota [25,26]. Specifically, treatment with 20(S/R)-ginsenoside Rh1 significantly reduced the *Firmicutes*/*Bacteroidetes* (F/B) ratio and decreased the abundance of the potentially harmful bacterial phylum *Desulfobacterota*. An elevated F/B ratio is known to exacerbate gut microbiota dysbiosis, thereby promoting colorectal carcinogenesis [27]. Analyses of multiple α-diversity indices and PCoA indicated that 20(S/R)-ginsenoside Rh1 treatment shifted the microbial community structure closer to that of the control group. Previous studies have reported that ginsenoside Rk3 can restore gut microbiota diversity and reduce the F/B ratio in CRC mice [28], which is consistent with our findings. More specifically, 20(S/R)-ginsenoside Rh1 can significantly increase the abundance of beneficial bacterial genera such as *Dubosiella*, *Muribaculum*, *Lactobacillus*, and *Limosilactobacillus*, while reducing the abundance of the carcinogenic bacterium *Streptococcus*. It has been reported that *Lactobacillus* and *Limosilactobacillus* are involved in Trp metabolism, alleviation of intestinal inflammation, repair of colonic mucosal damage, and anti-tumor activities [29]. Further findings of this study showed that 20(S/R)-ginsenoside Rh1 can significantly increase the abundance of *Lactobacillus johnsonii* and *Limosilactobacillus reuteri* in the gut. Both of which mediate Trp metabolism via specific enzyme systems to produce functional indole derivatives. Specifically, *Limosilactobacillus reuteri* utilizes indolepyruvate decarboxylase to catalyze the conversion of Trp into IAA [30]. Both aforementioned strains can independently produce IAld via aromatic amino acid transaminase (ArAT) [31], or synergistically generate IAld and ILA in conjunction with indole lactate dehydrogenase (ILDH) [32]. This suggests that 20(S/R)-ginsenoside Rh1 may directionally promote the conversion of tryptophan into active metabolites such as IAld, ILA, and IAA by enhancing the activity or expression of ArAT and indolepyruvate decarboxylase. Our experimental results demonstrated that 20(S/R)-ginsenoside Rh1 not only significantly increased the levels of indole derivatives (IPA, IAA, ILA, and IArA) in CRC mice, but also markedly activated the AhR, a key receptor for indole derivatives (Figure 10). Previous studies have shown that ginsenoside Rg1 can increase the levels of IAld, IPA, and ILA by enhancing the abundances of *Lactobacillus* and *Akkermansia* in ulcerative colitis mice [33], and that ginsenoside CK can alleviate colitis by activating AhR through increasing the concentrations of indoleacrylic acid (IA), IPA, and Indole-3-pyruvic acid [34]. Notably, the regulation of Trp metabolism by gut microbiota is not limited to the direct production of indole derivatives, but can also indirectly affect the Kyn pathway by regulating key nodes in host Trp metabolism [35,36]. In line with this, in CRC mice treated with 20(S/R)-ginsenoside Rh1, the Kyn concentration and the Kyn/Trp ratio were significantly reduced. Previous studies have indicated that Ginsenosides can decrease Kyn levels and the Kyn/Trp ratio in the plasma of depressed mice [37]. Importantly, excessive accumulation of kynurenine and an elevated Kyn/Trp ratio can induce the generation of Tregs, inhibit the production of effector T cells, lead to intestinal immune suppression, and promote immune escape of colon cancer cells [38].

Indole derivatives (such as IAA, IA, and ILA) have been confirmed to support the development and maintenance of IL-22 producing type 3 innate lymphoid cells (ILC3) in the intestine by activating AhR [39]. IL-22 activation can enhance intestinal barrier function and mucosal defense capabilities [40]. 20(S/R)-Ginsenoside Rh1 activates AhR by increasing the levels of IPA, IAA, ILA, and IArA, thereby promoting IL-22 production. Ginsenoside Rg1, on the other hand, activates the AhR/IL-22 signaling pathway by enhancing the level of 5-hydroxyindoleacetic acid to maintain intestinal barrier integrity [41]. In addition, 20(S/R)-ginsenoside Rh1 can significantly upregulate the expression of AhR downstream target protein CYP1A1, and the increased expression can further improve the transcriptional efficiency of intestinal IL-22 [42]. Consistent with our results, Ginsenoside CK can increase the expression of IL-22 in colon tissues of IBD mice by increasing the level of IA, activating AhR and upregulating CYP1A1 expression [34]. IPA can specifically activate PXR, upregulate the expression of intestinal tight junction proteins, and exert intestinal barrier protection function [43,44]. 20(S/R)-Ginsenoside Rh1 may activate PXR by increasing IPA levels, thereby upregulating the expression levels of intestinal tight junction proteins ZO-1, Claudin-1, and occludin in CRC mice. Some natural functional components also enhance intestinal barrier integrity by activating the PXR pathway [45], which suggests that activating PXR may be one of the common mechanisms by which natural products exert intestinal protective effects. Enzyme-linked immunosorbent assay (ELISA) results showed that 20(S/R)-ginsenoside Rh1 could downregulate the levels of pro-inflammatory factors TNF-α, IFN-γ, and IL-6, and upregulate the level of anti-inflammatory factor IL-10. The reason for the reduced inflammation level may be, on the one hand, the recovery of intestinal barrier function, which reduces the expression of pro-inflammatory factors. On the other hand, 20(S/R)-ginsenoside Rh1 inhibits the expression of TLR4 by activating PXR, reducing the release of pro-inflammatory factors. Notoginsenoside R1 reduces the levels of TNF-α and IL-6 by activating PXR receptors in colitis mice [46]. The above results suggest that 20(S/R)-ginsenoside Rh1 may reduce the risk of CRC by enhancing intestinal barrier integrity and inhibiting inflammatory responses.

The Trp-Kyn metabolic pathway is one of the important regulatory pathways for local immunosuppression in the tumor microenvironment (TME). Increased activity of this pathway is usually associated with Trp depletion and Kyn accumulation, which may subsequently contribute to the remodeling of the immunosuppressive microenvironment [47]. In the present study, we found that 20(S/R)-ginsenoside Rh1 significantly reduced the Kyn level and Kyn/Trp ratio. As the key rate-limiting enzyme in Trp metabolism, overexpression of IDO1 in the TME is the main cause of Trp depletion and Kyn accumulation [48,49], and our experimental results confirmed that 20(S/R)-ginsenoside Rh1 significantly downregulated the expression of IDO1. Overexpression of IDO1 can also promote the differentiation of CD4^+^ T cells into regulatory T cells (Tregs), ultimately leading to immune microenvironment imbalance and accelerated tumor immune evasion [50]. 20(S/R)-ginsenoside Rh1 significantly reduced the expression level of FoxP3, a marker of Treg cells, suggesting that Rh1 may reduce Treg cell differentiation by inhibiting the IDO1 pathway, thereby alleviating the immunosuppressive state of the TME. The pro-tumor effect of Kyn (an endogenous AhR ligand) catalyzed by IDO1 has been widely reported [14]. However, recent studies have found that ligands activating AhR are specific [51,52], and indole-3-carboxylic acid (an exogenous AhR ligand) can block kynurenine-mediated immunosuppression by competitively binding to AhR sites, significantly enhancing the anti-tumor activity of CD8^+^ T cells [52]. In addition, microbial indole derivatives can directly regulate CD8+ T cell function: ILA can activate CD8^+^ T cells, inhibit the expression of their cholesterol metabolism-related genes, and enhance the release of effector molecules, thereby killing CRC cells [53]; IPA promotes the differentiation of exhausted precursor CD8^+^ T cells into stem-like exhausted T cells by regulating histone acetylation, and this differentiation process can enhance the sustained killing ability of CD8^+^ T cells and the efficacy of immunotherapy [54]. The results of this study showed that 20(S/R)-ginsenoside Rh1 upregulated the number of CD8^+^ T cells in colon tumors, which was consistent with the effects of the aforementioned microbial indole derivatives, suggesting that it may enhance the anti-tumor immune activity of CD8^+^ T cells through the microbiota-mediated Trp metabolism pathway. Taken together, these results suggest that 20(S/R)-ginsenoside Rh1 may exert multi-layered regulation of Trp metabolism and immune cell function. On one hand, it inhibits the IDO1 pathway to reduce Kyn-mediated immunosuppression; on the other hand, it promotes the production of indole derivatives to enhance CD8^+^ T cell activity, providing experimental clues for further exploration of its regulatory role in the colorectal cancer immune microenvironment.

## 4. Materials and Methods

### 4.1. Preparation of 20(S/R)-Ginsenoside Rh1

20(S/R)-Ginsenoside Rh1 was prepared by microbial bioconversion of Ginsenoside Rg1 using *Lactiplantibacillus plantarum* TRG22. Following the protocol of Shen et al., [55]. Ginsenoside Rg1 (2 g L^−1^) was fermented at 37 °C for 21 days in a medium containing 30 g L^−1^ glucose and 10 g L^−1^ soybean oligopeptides. After fermentation, the broth was clarified by centrifugation (8000× *g*, 10 min) and loaded onto a D101 macroporous resin column. Sequential elution with water, 30% (*v*/*v*) ethanol, and 55% (*v*/*v*) ethanol removed impurities; the 55% ethanol fraction, which contained the target compounds, was pooled, concentrated under reduced pressure, and lyophilized. High-performance liquid chromatography (HPLC; Agilent 1260 Infinity II system, Agilent Technologies, Santa Clara, CA, USA) analysis was performed using a C18 column (250 mm × 4.6 mm, 5 μm; Waters, Milford, MA, USA) at 203 nm, resolving two well-separated peaks at 52.46 min and 53.27 min, corresponding to 20(S)- and 20(R)-Ginsenoside Rh1 standards, respectively. HPLC quantification confirmed the total purity of 95% for the final lyophilized product, with contents of 59.14% 20(S)- and 35.84% 20(R)-Ginsenoside Rh1. Liquid chromatography-mass spectrometry (LC-MS) (ESI^+^, *m*/*z* 639 [M+Na]^+^) and ^1^H, ^13^C nuclear magnetic resonance (NMR) spectra of the isolated material were identical to those reported for authentic 20(S/R)-ginsenoside Rh1, confirming identity and stereochemistry [56].

### 4.2. Animal Experimental

Male C57BL/6 mice (6-week-old, 18–20 g BW, specific pathogen-free (SPF) grade) were obtained from Liaoning Changsheng Biotechnology Co., Ltd. (Benxi, China). Animals were maintained in compliance with the guidelines approved by the Animal Care and Use Committee of Changchun University of Chinese Medicine. A total of 40 mice were randomly assigned to four groups (10 mice per group): control, model, Rg1, and Rh1 (Figure 1A). After a 1-week acclimation (Week 0), mice received a single intraperitoneal dose of azoxymethane (AOM, 10 mg kg^−1^, MP Biomedicals, Irvine, CA, USA) at the start of Week 1, except for the control group. Colorectal cancer was then induced with three 7-day cycles of 1.5% (*w*/*v*) dextran sulfate sodium (DSS, M.W. = 36,000–50,000, MP Biomedicals, Irvine, CA, USA) in drinking water (Weeks 2, 5, and 8), each followed by a 14-day recovery period with normal drinking water [57]. From Week 5 through Week 10, the Rg1 and Rh1 groups were given daily oral gavage of Ginsenoside Rg1 or 20(S/R)-ginsenoside Rh1 (100 mg kg^−1^ day^−1^) [58], respectively, whereas the control and model groups received vehicle (water) only. Disease Activity Index (DAI) was evaluated daily as described in [59]. Upon completion of euthanasia procedures, colonic specimens were subjected to morphometric assessment followed by longitudinal dissection for quantification of neoplastic lesions. Serum acquisition was achieved through centrifugation (6000 rpm, 10 min) conducted at 4 °C using collected whole blood. Colonic lumen contents and colonic tissues were harvested, flash-frozen in liquid nitrogen, and preserved at −80 °C for subsequent analysis.

### 4.3. Histological Examination

The paraformaldehyde-fixed (4%) colon tissues were dehydrated, cleared in xylene (2 × 10 min), embedded in paraffin, sectioned (5 μm thick), dried at 60 °C for 2 h, deparaffinized, and rehydrated before being stained with hematoxylin (5 min) and eosin (2 min). The histological changes in the colon tissues were visualized under an optical microscope (Olympus Corporation, Tokyo, Japan) at 200× magnification.

### 4.4. Biochemical Index Assay

Inflammatory cytokines IL-6, IL-10, TNF-α, and IFN-γ were quantified with ELISA kits (Jiangsu Meibiao Biotechnology Co., Ltd., Yancheng, China).

### 4.5. Gut Microbiota Analysis

Microbial genomic DNA was extracted from colonic lumen content samples using the QIAamp Fast DNA Stool Mini Kit (QIAGEN, Hilden, Germany). The V3–V4 region of the 16S rRNA gene was amplified with primers (F: 5′-ACTCCTACGGGAGGCAGCA-3′; R: 5′-GGACTACHVGGGTWTCTAAT-3′). PCR products were purified with QIAquick Gel Purification Kit (QIAGEN, Hilden, Germany) and sequenced on the Illumina NovaSeq platform (Illumina, Inc., San Diego, CA, USA). Following sequencing completion, all reads underwent quality assessment, with low-quality and truncated reads filtered out. Sequences were clustered into OTUs at a 97% similarity cutoff via USEARCH (version 10.0). OTUs were taxonomically assigned using the SILVA database (release 138.1; https://doi.org/10.5281/zenodo.4587955; accessed on 15 December 2025) and the Naive Bayes classifier in QIIME2 (70% confidence threshold). Alpha diversity was evaluated with QIIME2, whereas beta diversity was analyzed via principal coordinate analysis (PCoA) and Non-metric Multidimensional Scaling (NMDS). Microbial abundance differences were tested using one-way ANOVA, and differential taxa were identified through the LEfSe algorithm. Sequencing data were analyzed with the BMKCloud web platform (https://international.biocloud.net/; accessed on 15 December 2025).

### 4.6. Trp and Related Metabolites Quantified by LC-MS/MS

EXion LC Liquid Chromatography (AB SCIEX, Framingham, MA, USA) was used to evaluate serum Trp and its metabolites. With an injection volume of 5 μL and an Acquity UPLC HSS-T3 column (150 × 2.1 mm, 1.8 µm, Waters, Milford, MA, USA) thermostated at 40 °C, analyte separation was accomplished. Methanol and 0.1% formic acid made up mobile phase B, whereas water and 0.1% formic acid made up mobile phase A. 0–0.5 min, 10% phase B; 0.5–2 min, 10–30% phase B; 2–3 min, 60% phase B; 3–6 min, 60–98% phase B; 6–7.5 min, 98% phase B; 7.5–7.51 min, 98–10% B; and 7.51–9 min, 10% phase B were the elution gradients used for the study.

Metabolites were detected by mass spectrometry using an AB6500 Plus instrument (AB SCIEX, Framingham, MA, USA). The mass spectrometer parameters were configured as follows: an electrospray ionization (ESI) source was employed in positive ion mode, with the voltage set to 4500 V and the ion source temperature kept at 450 °C. Gas parameters comprised collision gas pressure at 10 psi, curtain gas pressure at 30 psi, and nebulizer and auxiliary gas pressure at 50 psi. Analysis of Trp, its metabolites, and the internal standard was carried out via multiple reaction monitoring (MRM).

### 4.7. Western Blotting

Tissue lysis buffer (Solarbio, Beijing, China, Cat. No. R0020) was used to make protein extracts from colon tissues, and the supernatant was collected after centrifugation. A bicinchoninic acid (BCA) protein assay kit (Coolaber, Beijing, China, Cat. No. SK1070) was used to measure and normalize the protein concentration. After undergoing electrophoretic separation, protein samples were put onto polyvinylidene fluoride (PVDF) membranes (Merck KGaA, Darmstadt, Germany, Cat. Nos. 03010040001) membranes. After blocking, primary antibodies against AhR, IL-22, occludin (Proteintech, Wuhan, China, Cat. Nos. 67785-1-Ig, 82766-15-RR, 27260-1-AP), PXR, TLR4 (Sangon Biotech, Shanghai, China, Cat. Nos. D122929, D264635), IDO1 (Beyotime Biotechnology, Shanghai, China, Cat. No. AF7161), ZO-1, and Claudin-1 (GeneTex, Irvine, CA, USA, Cat. Nos. GTX636491, GTX134842), CYP1A1(Bioss, Beijing, China, Cat. No. bs-1606R), and β-Actin (Servicebio, Wuhan, China, Cat. No. GB15003) were added to the membranes and incubated at 4 °C for the entire night. Following a 1-h incubation period with polyvinylidene fluoride (HRP)-conjugated secondary antibodies (Servicebio, Wuhan, China, Cat. No. GB23303) at room temperature, the membranes were chemiluminescently detected using an enhanced chemiluminescence (ECL) detection system (Clinx Science Instruments Co., Ltd., Shanghai, China).

### 4.8. Immunohistochemistry and Immunofluorescence

Colon tissues were fixed with 4% paraformaldehyde, embedded in paraffin, cut into 4 μm-thick sections, deparaffinized with xylene, and hydrated through graded ethanol. Antigen retrieval was then performed using 0.01 M citrate buffer (pH 6.0). Sections were treated with 3% hydrogen peroxide for 10 min at room temperature to inactivate endogenous enzymes, and blocked with 5% bovine serum albumin (BSA) at 37 °C for 30 min. For immunohistochemistry, sections were incubated at 4 °C overnight with the primary antibody against IDO1 (Beyotime, Shanghai, China, Cat. No. AF7161). Subsequently, HRP-conjugated goat anti-rabbit IgG secondary antibody (Servicebio, Wuhan, China, Cat. No. G1213) was applied, followed by incubation at room temperature for 1 h. Sections were treated with diaminobenzidine (DAB) solution for 3–5 min under microscopic monitoring to develop color, and hematoxylin was used as a counterstain. Photographs were taken using a brightfield microscope (Olympus Corporation, Tokyo, Japan). For immunofluorescence, sections were incubated at 4 °C overnight with primary antibodies against CD4 and CD8 (Abcam, Waltham, MA, USA, Cat. Nos. ab183685, ab316778). Secondary antibodies, one conjugated with Alexa Fluor 594 (for CD4, red fluorescence, Servicebio, Cat. No. GB28301) and the other with Alexa Fluor 488 (for CD8, green fluorescence, Servicebio, Wuhan, China, Cat. Nos. G1213, GB25303), were co-incubated with the sections for 1 h at room temperature. For FoxP3 single-labeling immunofluorescence, sections were incubated at 4 °C overnight with anti-FoxP3 primary antibody (Servicebio, Wuhan, China, Cat. No. GB150152). After thorough washing, sections were incubated with Alexa Fluor 594-conjugated secondary antibody (red fluorescence, Servicebio, Wuhan, China, Cat. No. GB28301) at room temperature for 1 h. 4′,6-diamidino-2-phenylindole (DAPI) was used for nuclear counterstaining, and images were captured using a laser scanning confocal microscope (Leica Microsystems, Wetzlar, Germany). Quantitative analysis of CD4^+^, CD8^+^, and FoxP3^+^ T cell counts and mean fluorescence intensity was performed using ImageJ software (version 1.54).

### 4.9. Reverse Transcription Quantitative PCR (RT-qPCR)

The Trizol reagent (Invitrogen, Carlsbad, CA, USA, Cat. No. 15596018CN) was used to extract total RNA from 50 mg of colon tissues ground in liquid nitrogen. RNA purity (OD_260_/OD_280_ = 1.8–2.0) and integrity were verified before downstream experiments. A cDNA synthesis kit (Servicebio, Wuhan, China, Cat. No. G3333) was used to reverse-transcribe 1 μg RNA into cDNA (20 μL system: 37 °C 15 min, 85 °C 5 s). RT-qPCR was carried out on a CFX Connect quantitative PCR system (Bio-Rad Laboratories, Hercules, CA, USA) using a 2× SYBR Green qPCR Master Mix (Servicebio, Wuhan, China, Cat. No. G3328) in a 20 μL system (10 μL Mix, 0.4 μL primers, 2 μL diluted cDNA (1:5), 7.2 μL water). Amplification program: 95 °C 30 s pre-denaturation, 40 cycles (95 °C 10 s, 60 °C 30 s), melting curve analysis for specificity. The housekeeping gene was *GAPDH*, and the 2^−ΔΔCt^ technique was employed to measure relative gene expression levels. The following primer sequences were employed for *IDO1*: (F: 5′-GAGGAGCAGACTACAAGAATGG-3′, R: 5′-GTGGATTTGGCAGAGCAAAG-3′) and *GAPDH*: (F: 5′-GGAGCGAGATCCCTCCAAAAT-3′, R: 5′-GGCTGTTGTCATACTTCTCATGG-3′).

### 4.10. Statistical Analysis

Data were analyzed with SPSS 20.0, ImageJ 1.54, Origin 2022, Visual Studio 2022 IDE, and GraphPad Prism 8.0. All data were expressed as mean ± standard deviation (SD). The experiment was divided into 4 groups with 10 mice per group. For each biological sample, technical replicates were performed in triplicate, and the average value was used for subsequent statistical analysis. For normally distributed data, statistical analysis was performed using one-way analysis of variance (ANOVA), followed by Fisher’s least significant difference (LSD) test. *p* < 0.05 was considered statistically significant.

## 5. Conclusions

This study suggests a correlation where 20(S/R)-ginsenoside Rh1 is associated with the regulation of Trp metabolism to produce indole derivatives by modulating the gut microbiota. These indole derivatives are linked to the activation of the AhR/CYP1A1/IL-22 and PXR/TLR4 pathways, which correlate with enhanced intestinal barrier function and reduced inflammation. In addition, 20(S/R)-ginsenoside Rh1 is significantly correlated with a reduction in the Kyn/Trp ratio, a change that may be associated with downregulated expression of IDO1. This effect appears to synergize with the gut microbiota-mediated Trp metabolic pathway and the regulation of immune cells in colon tumor tissues, collectively correlating with the alleviation of CRC. However, the specific mechanism by which metabolic regulation affects immune cells’ anti-tumor immunity remains unclear in this study, and future studies will explore the deep-seated correlations between gut microbiota, Trp metabolism, and anti-tumor immunity.

## Data Availability

The original contributions presented in this study are included in the article. Further inquiries can be directed to the corresponding authors.

## Abbreviations

The following abbreviations are used in this manuscript:

ArAT	Aromatic amino acid transaminase
AhR	Aryl hydrocarbon receptor
CYP1A1	Cytochrome P450 family 1 subfamily A member 1
IAA	Indoleacetic acid;
IA	Indoleacrylic acid
IArA	Indole-3-acrylic acid
IDO1	Indoleamine 2,3-dioxygenase 1
IFN-γ	Interferon gamma
ILA	Indole-3-lactic acid
IL-22	Interleukin-22
IL-6	Interleukin-6
IL-10	Interleukin-10
IPA	Indole-3-propionic acid
Kyn	Kynurenine
KynA	Kynurenic acid
PXR	Pregnane X receptor
TLR4	Toll-like receptor 4
TME	Tumor microenvironment
Tregs	Tregulatory T cell
TNF-α	Tumour necrosis factor-alpha
Trp	Tryptophan
